# An 80-year-old female with multiple mononeuropathy: neuroleukemiosis a rare complication of chronic lymphocytic leukaemia

**DOI:** 10.1093/omcr/omaf131

**Published:** 2025-08-11

**Authors:** Caroline Morgan, Venkataramanan Srinivasan, Ute Pohl, Benjamin R Wakerley

**Affiliations:** University Hospitals Birmingham NHS Foundation Trust, Mindelsohn Way, Edgbaston, Birmingham, B15 2WB, United Kingdom; University Hospitals Birmingham NHS Foundation Trust, Mindelsohn Way, Edgbaston, Birmingham, B15 2WB, United Kingdom; University Hospitals Birmingham NHS Foundation Trust, Mindelsohn Way, Edgbaston, Birmingham, B15 2WB, United Kingdom; University Hospitals Birmingham NHS Foundation Trust, Mindelsohn Way, Edgbaston, Birmingham, B15 2WB, United Kingdom; University of Birmingham, Wolfson Drive, Edgbaston, Birmingham, B15 2TT, United Kingdom

**Keywords:** chronic lymphoid leukaemia, neuroleukemiosis, multiple mononeuropathy

## Abstract

We report the case of an 80-year-old lady with chronic lymphocytic leukaemia who presented with painful footdrop. Nerve conduction studies and electromyography confirmed the presence of multiple mononeuropathy. Left superficial radial nerve biopsy revealed small lymphocytes infiltrating the epineurium and perineurium and confirmed a diagnosis of neuroleukemiosis.

## Introduction

Neuroleukemiosis, defined as the infiltration of leukaemia in the peripheral nerve structures [1], is an extremely rare complication in leukaemia and when present, more common in acute leukaemia compared to chronic lymphocytic leukaemia (CLL). Decision for treatment of CLL depends on staging criteria and signs and symptoms of active disease [2]. Patients who are Rai stage 0 (low risk) or Rai stage I or II (intermediate risk) are often monitored without treatment.

Here, we report a case of neuroleukemiosis in CLL, causing multiple mononeuropathy.

## Case presentation

An 80-year-old lady had a diagnosis of CLL—under 6 monthly surveillance. Rai stage I due to enlarged lymph nodes. She presented 20 months after diagnosis with painful left foot drop and left foot paraesthesia. She initially experienced paraesthesia in the right-hand fingertips 12 months following diagnosis. This was followed by reduced sensation on the dorsum of the right foot and pain. She then experienced left hand paraesthesia in the fingertips and inability to do fine motor tasks due to weak grip strength. At 18 months post diagnosis, she developed left painful foot drop which progressively worsened, prompting neurology assessment. Examination revealed reduced vibration sense in left thumb, power in left ankle dorsiflexion and toe extension 0/5, but normal eversion, inversion, and plantar flexion. On the right side, abductor hallucis was significantly weak although plantar flexion was reasonably well preserved. Submandibular lymphadenopathy was present with no organomegaly.

### Investigations

B12, folate and HbA1c were normal. Blood borne virus screen was negative. Vasculitis screen revealed raised Myeloperoxidase- ANCA antibodies. Cerebrospinal fluid was unremarkable and flow cytometry revealed no evidence of clonal lymphoid cells. MRI head and whole spine revealed no abnormalities. CT Thorax-Abdomen-Pelvis revealed extensive thoracic, abdominal, pelvic and groin adenopathy in keeping with a lymphoproliferative disorder. PET-CT revealed mildly avid lymphadenopathy on both sides of the diaphragm without FDG avid bone or splenic involvement.

Nerve conduction studies (Tables 1 and 2) and electromyography (Table 3) were suggestive of bilateral median nerve dysfunction at the level of the wrist and ulnar nerve dysfunction across the left elbow and an axonal lesion of the left median nerve. All the motor and sensory responses distal to the ankle were significantly reduced on the right side with more pronounced ongoing denervation activity in the right foot muscles as compared to the left foot muscles or proximal tibial nerve-innervated muscle. Left superficial peroneal sensory and motor response from left TA response was significantly reduced on the left side, ongoing denervation activity and chronic neurogenic changes were noted in a patchy manner in left lower limb muscles. In summary, the findings supported a patchy asymmetric sensory-motor axonal neuropathy specifically in the clinically symptomatic regions of the lower limbs i.e. left hand, left common peroneal nerve and distal segment of the right tibial nerve (distal to ankle). This raised a strong possibility of multiple mononeuropathy secondary to leukemic infiltration.

**Table 1:** Nerve conduction study/sensory.

Sensory nerve	Lat	Amp	CV
	ms	μV	m/s
CTS Sensory Left			
Med. Index—Wrist	2.59	1.17	44.4
CTS Sensory Right			
Med. Index – Wrist	2.75	4.6	41.8
Dig plant med Sensory Left			
Sole – Ankle	2.65	3.1	—
Dig plant med Sensory Right			
Sole – Ankle	—	NR	
Peroneus superficial Sensory Left			
Lower leg – Ankle	2.00	4.2	50.0
Peroneus superficial Sensory Right			
Lower leg – Ankle	1.44	20.3	59.0
Suralis Sensory Left			
Mid. Lower leg – Ankle	1.92	15.9	49.5
Suralis Sensory Right			
Mid. Lower leg – Ankle	1.85	17.5	48.6

**Table 2:** Nerve conduction study/motor.

Nerve	Lat	Amp	CV	Dur
	ms	mV	m/s	ms
Median (Lumbricals) Motor Left				
Median(wrist)—2nd Lumbrical	4.15	0.63		4.0
Median (Lumbricals) Motor Right				
Median(wrist)—2nd Lumbrical	3.86	1.29		4.8
Medianus Motor Left				
Wrist—APB	4.59	1.34		6.1
Medianus Motor Right				
Wrist—APB	4.19	7.0		4.5
Peroneal TA Motor Left				
Fib. Head—TA	5.77	0.47		9.4
Popliteal Fossa-Fib. Head	9.95	0.14	12.0	10.2
Peroneal TA Motor Right				
Fib. Head—TA	2.77	10.1		13.4
Peroneus Motor Left				
Ankle—EDB	3.79	6.1		6.0
Fib. head-Ankle	10.9	5.7	43.6	6.5
Peroneus Motor Right				
Ankle—EDB	—	—		—
Fib. head-Ankle	—	—	—	—
Tibialis Motor Left				
Ankle—Abd hal	4.78	9.8		5.0
Knee-Ankle	5.13	9.6	—	4.9
Tibialis Motor Right				
Ankle—Abd hal	10.7	0.28		4.5
Ulnar FDI Motor Left				
Wrist—FDI	3.85	5.6		5.4
Bl. Elbow-Wrist	8.76	2.9	41.8	5.4
Ab. Elbow-Bl. Elbow	11.6	2.9	28.2	6.1
Ulnar FDI Motor Right				
Wrist—FDI	3.71	5.0		4.5
Bl. Elbow-Wrist	8.60	4.6	41.9	4.2
Ab. Elbow-Bl. Elbow	10.5	4.7	42.1	4.6

**Table 3:** Electromyography.

Muscle	IA	Fib	PSW	Fasc	Amp	Dur	Poly	Recruit
Right Abd dig min		2+	2+	None	+	+	None	Reduced
Left Abd dig min		1+	1+	None	+	+	Few	Reduced
Right Adductor hallucis		2+	2+	None	+	+	None	Reduced
Left Flex dig longus		1+	2+	None	+	+	Few	Reduced
Right Gastroc (med)		None	None	None	N/+	N/+	Few	Reduced
Left Gastroc (med)	Incr	None	None	None	+	+	Few	Reduced
Right Peroneus longus		None	None	None	+	+	Many	Reduced
Left Peroneus longus		1+	1+	None	+	+	None	Reduced
Right Tibialis anterior		None	None	None	+	+	Many	Reduced
Left Tibialis anterior		2+	2+	None	−	−	−	−

*Tables 1,2,3*: *DML = distal motor latency; CMAP = compound muscle action potential; CV = conduction velocity; SNAP = sensory nerve action potential. —, values not recorded; L = Left; R = Right; CTS = Carpal Tunnel Syndrome; Fib = Fibular; dig plant med = digital plantar medial nerve; APB = Abductor policis brevis TA = Tibilais anterior; EDB = Extensor digitorum brevis; FDI = First Dorsal interosseous; IA = insertion activity; Fib = fibrilation potential: PSW = Positive Sharp Waves; Fasc = Fascciulation potentials; Amp = amplitude; Dur = duration;Poly = polyphasic potentials; Recruti = recruitment pattern; Abd dig min = Abductor digiti minimi pedis; Fle dig longus = Flexor digitorum longus; Gastroc = gastrocnemius; Inc = increased*

**Figure 1:**
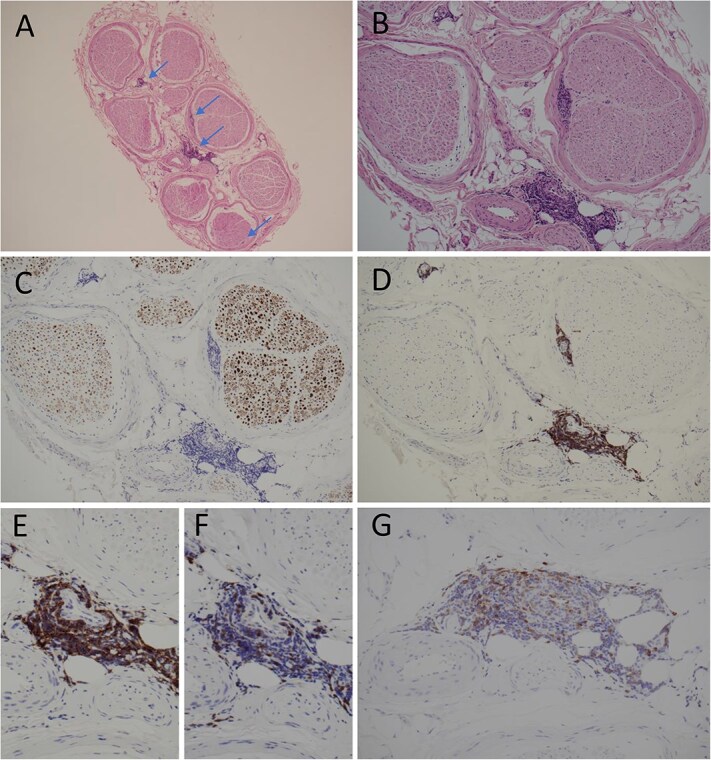
Histology of transected superficial radial nerve involved by CLL. (A and B); HE stains showing multifocal lymphocytic infiltrates (arrows; ×40 and ×100 mag. Respectively); (C), immunohistochemistry (IHC) for neurofilament reveals mild reduction of axons (x100 mag.); (D and E), IHC for CD20 highlights predominant B-cell component of perivenular infiltrates (×100 and ×200 mag., respectively); (F), IHC for CD3 shows scanty positive cells compatible with reactive T-cells (×200 mag.); (G), IHC reveals weak to moderate CD5 positivity in the majority of B-cells (x200 mag.) with coexpression of CD23 (the latter is not illustrated).

Left superficial radial nerve biopsy revealed neither vasculitis nor significant axonal or demyelinating neuropathy. However, the epineurium and perineurium showed a patchy perivascular infiltration by small lymphocytes with a predominant B-cell component which stained positive for B-cell marker CD20, CD5 and CD23 (Fig. 1). The appearances were diagnostic of CLL infiltrating the peripheral nerves.

## Discussion

Neuroleukemiosis in CLL is rare, with only 6 previous reports in the literature [3–7]. Of these case reports, five already had a diagnosis of CLL prior to identification of neuroleukemiosis [3–6] and one had neuroleukemiosis as the clinical presentation for CLL [7]. Four cases were identified by nerve biopsy [3, 5–7], one identified by a mass lesion on MRI, which further revealed avid uptake on PET [5] and one identified by autopsy [4].

Nerve biopsy remains the gold standard for confirmation of peripheral nerve infiltration. However, the results may be negative or non-specific due to the patchy nature of infiltration. Cases have been reported with negative sural nerve biopsy, despite sural nerve involvement, suggesting the focal infiltration of the nerve and requirement of biopsy of the specifically affected part of the nerve [8]. Case reports have suggested that a target for biopsy may be revealed by positive MRI or PET-CT findings [9]. However, in this case MRI and PET-CT were negative so the target for biopsy was identified by the neurophysiology results. The sural nerve was of normal amplitude so the superficial radial nerve was chosen, and biopsy was taken under the hand surgical team so an allograft could also be done.

In this case, the patient had a diagnosis of CLL, Rai stage 1 and was under 6 monthly surveillance. Her initial neurological symptoms of paraesthesia in the right hand started one year after her diagnosis of CLL. However, investigations for her progressively worsening peripheral neuropathy were 20 months after diagnosis of CLL. The initiation of chemotherapy shows positive recovery of neurological symptoms in previous case reports [3]. The patient had no negative haematological predictive factors, thus not requiring treatment. However, the rare complication of nerve infiltration challenges this notion and suggests these rare complications should be screened at diagnosis and during surveillance to initiate prompt treatment and avoid progression of disease.

## Conclusion

Neuroleukemiosis is a rare complication of CLL. Clinicians should consider this diagnosis in patients with a new diagnosis of leukaemia or history of leukaemia who present with peripheral nerve damage suggestive of multiple mononeuropathy. Early recognition, diagnosis and treatment of this rare complication may reduce progression of neurological deficits. Nerve biopsy is invaluable in the accurate diagnosis of neuroleukemiosis and should be considered when clinical suspicion is high.

## Data Availability

Data on request from the authors.

## References

[1] Aregawi DG, Sherman JH, Douvas MG. et al. Neuroleukemiosis: case report of leukemic nerve infiltration in acute lymphoblastic leukemia. Muscle Nerve 2008;38:1196–200. 10.1002/mus.21089 18642385

[2] Hallek M, Cheson BD, Catovsky D. et al. iwCLL guidelines for diagnosis, indications for treatment, response assessment, and supportive management of CLL. Blood 2018;131:2745–60. 10.1182/blood-2017-09-806398 29540348

[3] Vicino A, Cochet S, Pistocchi S. et al. A severe case of neuroleukemiosis caused by B cell chronic lymphocytic leukemia, presenting as mononeuritis multiplex. J Peripher Nerv Syst 2023;28:266–8. 10.1111/jns.12552 37119473

[4] Grisold W, Jellinger K, Lutz D. Human neurolymphomatosis in a patient with chronic lymphatic leukemia. Clin Neuropathol 1990;9:224–30. 2272142

[5] Reddy CG, Mauermann ML, Solomon BM. et al. Neuroleukemiosis: an unusual cause of peripheral neuropathy. Leuk Lymphoma 2012;53:2405–11. 10.3109/10428194.2012.691480 22571477

[6] Sommer C, Carroll AS, Koike H. et al. Nerve biopsy in acquired neuropathies. J Peripher Nerv Syst 2021;26:S21–41. 10.1111/jns.12464 34523188

[7] Briani C, Visentin A, Cavallaro T. et al. Primary neurolymphomatosis as clinical onset of chronic lymphocytic leukemia. Ann Hematol 2017;96:159–61. 10.1007/s00277-016-2852-2 27761605

[8] van den Bent MJ, de Bruin HG, Bos GM. et al. Negative sural nerve biopsy in neurolymphomatosis. J Neurol 1999;246:1159–63. 10.1007/s004150050535 10653308

[9] Le Clech L, Rizcallah MJ, Alavi Z. et al. Mononeuritis mult iplex in a patient with B-cell prolymphocytic leukaemia: a diagnostic challenge. BMJ Case Rep 2013;2013:bcr2013009425. 10.1136/bcr-2013-009425 PMC3794323 24000206

